# Longevity of Plant Pathogens in Dry Agricultural Seeds during 30 Years of Storage

**DOI:** 10.3390/microorganisms9102175

**Published:** 2021-10-19

**Authors:** Guro Brodal, Åsmund Asdal

**Affiliations:** 1 Norwegian Institute of Bioeconomy Research (NIBIO), P.O. Box 115, NO-1431 Ås, Norway; 2 Nordic Genetic Resource Center (NordGen), P.O. Box 41, SE-230 53 Alnarp, Sweden; asmund.asdal@nordgen.org

**Keywords:** seed-borne diseases, long-term seed storage, seed health, germplasm, 100-year storage experiment, Svalbard

## Abstract

Plant diseases may survive and be spread by infected seeds. In this study we monitored the longevity of 14 seed-borne pathogens in 9 crop species commonly grown in the Nordic countries, in addition to a sample of sclerotia of *Sclerotinia sclerotiorum*. The data from the first 30 years of a 100-year seed storage experiment located in a natural −3.5 °C environment (permafrost) in Svalbard, Norway, are presented. To date, the pathogens, tested by traditional seed health testing methods (freezing blotter, agar plates, growing on tests), have survived. Linear regression analyses showed that the seed infection percentages of *Drechslera dictyoides* in meadow fescue, *Drechslera phlei* in timothy, and *Septoria nodorum* in wheat were significantly reduced compared to the percentages at the start of the experiment (from 63% to 34%, from 70% to 65%, and from 15% to 1%, respectively), and that *Phoma betae* in beet had increased significantly (from 43% to 56%). No trends in the infection percentage were observed over the years in *Drechslera* spp. in barley (fluctuating between 30% and 64%) or in *Alternaria brassicicola* in cabbage (fluctuating between 82% and 99%), nor in pathogens with low seed infection percentages at the start of the experiment. A major part of the stored sclerotia was viable after 30 years. To avoid the spread of seed-borne diseases, it is recommended that gene banks implement routines that avoid the use of infected seeds.

## 1. Introduction

Many plant species, including food and feed crops, are propagated by seeds. To maintain and ensure the availability and diversity of plant genetic resources, the seeds of many crop species are collected, dried to around 4–7% moisture content, and conserved in sealed moisture-proof containers, e.g., glass ampoules, plastic containers, or aluminum foils for long-term storage [1] in gene banks under cold conditions. To mitigate the risk of loss during long-term storage caused by, e.g., poor storage and handling conditions and natural or man-made disasters, safety duplicates (back-ups) of seed collections have been established [2,3]. In 1984, the Nordic Gene Bank (NGB, now NordGen), established a duplicate collection of Nordic seed accessions in a natural −3.5 °C environment (permafrost) in an abandoned coal mine in Svalbard, a Norwegian archipelago in the Arctic Ocean [4]. Two years later, the NGB started a seed storage experiment located in the same storage facilities. [4,5]. The storage experiment, containing seeds of agricultural and horticultural crop species commonly grown in the Nordic countries, is intended to last for 100 years and is known as the 100-year experiment. The purpose of the experiment is to monitor the seed germination, moisture content, and longevity of pathogens in dry seed material (dried to 3–5% moisture content at the start of the experiment) sealed in glass ampoules under permafrost conditions.

The seeds of most crops carry a wide variety of pathogens that can survive from a few months to several years on or inside them [6]. The longevity of these seed-borne pathogens during storage have not been widely studied in recent years; however, earlier studies have shown that survival depends largely on the moisture content of the seed, the storage conditions (temperature and moisture), and host species, as summarized by Agarwal and Sinclair [7]. Moreover, the duration of survival varies among pathogens and is influenced by the amount of inoculum on each seed, the type of survival structures (e.g., hyaline/fragile, or pigmented/thick-walled spores, mycelia, and fruiting bodies), and the location of inoculum in the seeds. Conditions favourable for seed longevity usually also favour pathogen survival. In addition, examples of pathogens that may live even longer than the seeds they colonize have been reported [7]. In general, the storage of seeds under dry and cool conditions is known to maintain the viability of seed-borne inoculum, although survival will decrease with increased storage duration.

The risk of spreading pathogens through infected seed exchange is well known, and gene banks need to take measures to minimize the risk of spread by infected germplasm [8]. The storage of seeds has in a few cases been used as a method to eliminate seed-borne fungi; however, it is considered too variable and unreliable to be used as a control method [9]. Additional knowledge on the survival of seed-borne pathogens under dry and cool conditions would be useful for gene banks.

The results for germination and moisture content of the seed samples during the first 30 years of the 100-year NGB seed storage experiment in permafrost were recently summarized [10]. In this paper we report on the longevity of seed-borne pathogens in the seed samples selected for the study of pathogen survival. Our hypothesis was that seed infection percentages during the first 30 years would not decline. Based on our current data, this hypothesis had to be rejected for most of the pathogens. However, all seed-borne pathogens have survived until now. We discuss and compare our results with data from previous studies on the longevity of seed-borne pathogens during seed storage.

## 2. Materials and Methods

### 2.1. Storage Facilities

The 100-year seed storage experiment, established in 1986, is being performed in an abandoned transverse passage of a coalmine outside Longyearbyen (78 °N), Svalbard, Norway. Seeds, dried to 3–5% moisture content and sealed in glass ampoules, are stored in a steel container placed approximately 285 m from the entrance, 200 m above sea level, under 70 m of solid rock. The permafrost keeps the temperature in the transverse passage at approximately −3.5 °C all year round and makes the storage independent of energy input. When the Svalbard Global Seed Vault (www.seedvault.no, accessed on 20 September 2021) was opened in 2008, the NBG-duplicated seed accessions were transferred to the Vault. However, the material from the 100-year experiment was kept in the coal mine passage.

### 2.2. Seed Materials and Sample Preparation

The longevity of seed-borne pathogens was studied in 9 naturally infected crop plant species represented by 1 seed lot per species, except for wheat, where 2 seed lots were included. The host crops, their pathogens, and the origin of the seed materials are presented in Table 1. After drying the seeds to approximately 3–5% moisture content, 25 samples of 2 × 500 seeds from each seed lot were sealed in glass ampoules [4,10]. In addition, samples of dry sclerotia (fungal survival structures) of *Sclerotinia sclerotiorum* collected from a Norwegian cabbage seed lot were conserved in ampoules (20 sclerotia in each) without any seed material. Thus, 1 sample from each seed lot, and the sclerotia lot, were packed in a wooden box. Altogether, 25 boxes (i.e., 1 box for each analysis event which was planned every 2.5 years during the first 20 years and every 5 years for the next 80 years), each containing 1 series of samples, were prepared and transported to Longyearbyen, Svalbard, and placed in the storage container. The first series of samples were analysed at the start of the experiment in December 1986 (year 0) and the most recent analyses were completed in December 2016 (year 30), i.e., 11 seed analysis events to date. The last analysis event will be in December 2086 (year 100).

### 2.3. Pathogen Analyses

Seeds of the included crop species, except lettuce with lettuce mosaic virus (LMV), have been analysed for fungal pathogens at Kimen Seed Laboratory, Ås, Norway, an accredited International Seed Testing Association (ISTA) member laboratory. Most pathogen species, including *Septoria nodorum*, *Drechslera* spp., *Fusarium* spp., *Botrytis allii,* and *Alternaria* spp., were analysed by an internal ‘freezing blotter method’ (FBM) (Table 1) based on protocols in the literature [11,12] and relevant ISTA ‘Working sheets’ available at the start of the experiment [13,16,17,19]. With this method, seeds were evenly plated on moist filter paper (blotters) in transparent polystyrene dishes and incubated for 24 h at room temperature followed by freezing (−20 °C) for 24 h and subsequently 5 days at 20 ± 2 °C with alternating 12 h near-ultraviolet (NUV) light and 12 h darkness. The beet sample was analysed for *Phoma betae* by plating seeds on artificial growth substrate (water agar (WA) in Petri dishes) (Table 1), and was incubated for 1 week at 20 °C in darkness [18]. Although the taxonomic nomenclature has been updated with new names for some of the pathogens, we maintained the scientific names given in the instructions with the samples in the storage boxes. Of each sample, 400 seeds were analysed at each testing event. Identification of the fungal pathogens was based on the characteristics of spores and fruiting bodies developed on seed and on the substrate (paper or agar) during incubation. The examination was done using a stereomicroscope at 50× and sometimes by examining microscopic slides using a microscope at higher magnifications (100–400×). The numbers of seeds infected by the investigated pathogens were recorded and the seed infection percentages with regard to each pathogen were calculated from the number of seeds tested.

The analysis for *Ustilago nuda* f. sp. *tritici*, causing loose smut in wheat, was conducted by a symptom method (SM, also called ‘growing-on’ method, Table 1) by sowing 1000 seeds into a standard soil (60% peat/40% clay) in the greenhouse at 10 °C until emergence, and thereafter by growing the plants at 15 °C (16 h day/8 h night) until heading. The number of plants showing loose smut symptoms at heading were recorded and the percentage of infected plants (indicating viable seed infection) was calculated from the total number of emerged plants.

The analysis for LMV was conducted at the Norwegian Institute of Bioeconomy Research (NIBIO) in Ås, Norway, also using the SM method (Table 1), by sowing 600 seeds into a standard soil (60% peat/40% clay) in the greenhouse at approximately 18–20 °C (16 h day/8 h night). The emerged plants were assessed for symptoms of LMV in the first 3 well-developed leaves [14]. All plants showing mosaic symptoms as well as plants with weak signs of infection were tested separately for LMV using the ELISA method [15]. Plants testing positive with the ELISA method were recorded and the percentage of infected plants (indicating viable seed infection) was calculated from the total number of emerged plants.

The viability of 20 sclerotia (*S. sclerotiorum*) was analysed by plating them on potato dextrose agar (PDA) in petri dishes, 1 sclerotium per dish, after surface disinfection in NaOCl (1% available Cl) for 10 min and incubation at 20 °C for 1 week. The number of sclerotia showing rapid growth of white cottony mycelium was recorded.

The same methods as described above will be used throughout the whole 100-year seed storage experiment to assess survival of seed-borne pathogens. A copy of the method procedures is included in each box/series of samples.

### 2.4. Statistical Analyses

The seed infection data of each pathogen (except *S. sclerotiorum*) from the 11 analysing events were tested for a possible trend in the seed infection percentages over the years by simple linear regression using Minitab 18.

## 3. Results and Discussion

All 15 pathogens (including *Fusarium* in 3 different crops) studied in the 100-year storage experiment in permafrost survived the first 30 years and all were detected at the 10 testing events after initiation, except for *Fusarium* in onion which was not detected at 17.5 years after the start of the experiment (Table 2). The initial seed infection percentages varied considerably among the different crops, from only 1 or several % to up to 90% infection. Simple linear regression analyses revealed that infection percentage of 3 of the investigated pathogens (*Drechslera dictyoides*, *Drechslera phlei* and *Septoria nodorum*) declined significantly and that 1 pathogen (*Phoma betae*) had increased over 30 years of storage. Among the other pathogens, no significant trends were found; however, the infection percentages fluctuated during the years (Table 2). Despite efforts to obtain samples that were representative of the seed lots, some random sampling variation between sub-samples during preparation of the materials for the experiment cannot be excluded. Infection by pathogens was shown to result in greater variation between tests than was expected from theoretical sampling error (e.g., [12]), and it is likely that this contributed to the differences between testing events in our study. Moreover, a slight change in the state of the stereomicroscope or in the examiners’ concentration on the work may lead to variations in results between analysis events.

### 3.1. Reduced Seed Infection Percentages during Storage

Seed infection by *D. dictyoides*, causing net blotch/leaf blight/leaf spot in meadow fescue, seemed to decline after 5 years, and was reduced from 63% to 34% (*p* = 0.00) during the storage period (Table 2). The infection percentage of *D. phlei*, causing leaf spot/leaf streak in timothy, was reduced from 70% to 65% (*p* = 0.01) after 30 years, with an indication of reduced longevity after 12.5 years. In a previous study of *Drechslera* spp. in Norwegian timothy and meadow fescue seed the infection percentages were considerably reduced after 3 years of storage at room temperature, from 12% to 6% on average for 12 meadow fescue samples and from 24% to 10% on average for 16 timothy samples [20]. A reduced incidence of *Drechslera* spp. in ryegrass seed during storage at room temperature was also reported in New Zealand [21]. However, in a Canadian study, *D. phlei* was isolated from seed of *Phleum nodosum* after 7 years at −20 °C [22]. This is in line with our results that *Drechslera* spp. in grass seed may survive for several years at temperatures below zero, despite some decline in viability percentage during storage.

*Septoria nodorum*, causing glume and leaf blotch in wheat, showed reduced longevity, from 15% to 1% (*p* = 0.02) during the first 30 years of our storage experiment, although some variation was observed among testing events (Table 2). In contrast, a study from the United Kingdom reported a decline from 50% to 39% infection after 14 years of storage at −20 ° [23]. Moreover, in the United States the presence of the pathogen has been observed to increase considerably (from around 50% to over 70% infected seeds) during storage at both 5 and 25 °C over 2 years after harvest, with a more rapid infection increase in seed lots stored at 25 °C than at 5 °C [24]. It was suggested that the increase in detection of *S. nodorum* was caused by decline of other more fast-growing seed-borne fungi, especially *Alternaria* and *Epicoccum*. A continued study with the same seed lots [25] reported that regardless of annual variations, the survival of *S. nodorum* was still high after 10 years at 5 °C (at the same level as detected after harvest); however, viability was lost within 3–4 years in seeds stored at 25 °C. On the other hand, *S. nodorum* seed infection declined from 25–30% to 0–5% after a year in a German study, and the decline was more rapid at high (25 and 30 °C) compared to low (4, 10, and 15 °C) storage temperatures [26]. A Canadian study reported that *S. nodorum* in wheat declined rapidly the first 4 years but was still detected at low levels after 7 years of storage of seeds kept in paper envelopes in a metal box in an unheated dry shed [27]. Despite varying results for longevity of *S. nodorum* in wheat seed, it can be concluded that this pathogen survives for many years when stored at low or below zero temperatures such as in germplasm collections.

### 3.2. Increased Seed Infection Percentage during Storage

*Phoma betae*, causing blackleg and damping-off in beet, showed an increased infection level after 30 years compared to the level at the start of the experiment (from 43% to 56%, *p* = 0.02). The increased infection percentages were observed towards the last years of storage, whereas only slight variation was seen until year 15 (Table 2). With the WA method, *P. betae* develops hyaline hyphae that grow towards the bottom of the dish where they form swelling structures referred to as holdfasts [18]. According to the method description holdfast developments may be restricted in the presence of bacteria. One explanation for the increased infection percentage might be disappearance of putative bacteria, or other perhaps inhibiting seed-borne fungi, facilitating the growth of *P. betae* in the agar. Neither bacteria nor other fungi beside *P. betae* were included in the analyses and were therefore not recorded during the examination of the samples. However, increased infection percentages during seed storage have been observed for other seed-borne fungi, e.g., *S. nodorum,* explained by the decline of other more fast-growing seed-borne fungi [24]. In contrast to our findings, studies in the United Kingdom reported a reduction from 30% to 23% of *P. betae* infected seed after 14 years of storage of sugar beet at −20 °C [23], and a reduced infection percentage from 27.5% to 4.5% in beet seeds stored for 13 years at 10 °C and 50% RH [28]. However, the latter study showed a large variation between the yearly results of *P. betae* (between 14% and 35% within 3 years).

### 3.3. No Change in Seed Infection Percentages during Storage

The seed infection percentages of *Drechslera* spp., causing net blotch/spot blotch and leaf stripe disease in barley, did not show a significant trend in the infection level over 30 years. However, the level of infected seeds fluctuated considerably, from 38% at the start to 64% after 5 years and to 30% after 25 years (Table 2). An increased infection percentage during storage was previously reported in a Canadian study, where *D. teres* in barley increased from around 3% to around 8% infected seeds during the second and the third year of storage, despite storage in an aerated box in an unheated dry shed [27]. In the same study, infection with *D. avenae* in oat seeds increased during the first 5 years of storage from around 20% to 45%. A suggested reason for the increased infection frequencies was that during the first few years of storage there might have been saprophytic microorganisms present that inhibited or covered the growth of *D. teres*. However, after 10 years the infection levels in the Canadian barley and oat seeds were reduced to 3% and 10% infected seeds, respectively. A reduction from 24% to 16% of *D. teres*, and from 47% to 44% in *D. graminea* on barley seeds stored at −20 °C for 12 years was reported in a study from the United Kingdom [23]. A more recent evaluation of seed-borne fungi (including *Drechslera* spp.) in 36 rye accessions after 30 years of storage at −4 °C in the national Spanish gene bank did not reveal any strong effect of storage on seed infection rates [29], which is generally in accordance with our results. Together, this indicates that *Drechslera* spp. can survive a long time in dry cereal seeds stored at temperatures below zero degrees.

A high infection percentage of *A. brassicicola*, causing dark leaf spot in cabbage, was maintained over the years; 90% infected seeds at the start and 84% after 30 years (Table 2). A survey of seed-borne fungi on genebank-stored (−1 °C, 30% RH) cruciferous seeds in Japan showed that the seed lots were frequently infected by pathogenic fungi such as *A. brassicicola* after around 10 years of storage [30]. This is in line with the high level of *A. brassicicola* on cabbage seeds that we observed in our experiment during the 30 years of storage in permafrost. A study in the United Kingdom reported a clear reduction in the viability of surface-borne spores and mycelium of *A. brassicicola* on cabbage seeds after 2 years of storage at 10 °C and 50% RH [31], indicating a low importance of these structures as inoculum source. However, internal infection persisted for up to 12 years in that study.

Low infection percentages at the start of the experiment were recorded for *Fusarium* in barley (8%), wheat (5%) and onion (1%), *U. nuda* f. sp. *tritici* (5.2%) in wheat, *Alternaria dauci* (6%) and *A. radicina* (4%) in carrot, *Botrytis allii* (4%) in onion, and LMV (1.8%) in lettuce (Table 2). No significant changes in the infection levels for these pathogens were observed after 30 years, although some fluctuations in the levels were seen between testing events, e.g., *Fusarium* in barley and wheat, and *B. allii* in onion.

*Fusarium* spp. are known to decline rapidly (after few months) in cereal seed stored at room temperature e.g., [32,33,34]. However, in our experiment no reductions in *Fusarium* levels in barley, wheat and onion seed were observed (Table 2). We do not know if some of the infections recorded as *Fusarium* in barley and wheat might belong to *Microdochium nivale/majus* which previously was denominated *Fusarium nivale*. Morphologically it can be difficult to differentiate between the genera *Fusarium* and *Microdochium* with the method that was used because it does not always promote sporulation. At the time of the collection of the materials for the experiment, *Microdochium* were more prevalent in cereal seeds than those species that are denominated as *Fusarium* today. If it was *Microdochium* in the barley and wheat seeds in our experiment, the maintenance of the infection level is in accordance with the findings of a study in the United Kingdom which detected 19% infection of *Microdochium* in barley and wheat both at the start and after 12 years of storage at −20 °C [23]. At the next testing event in our experiment a more precise diagnosis of *Fusarium* spp. in barley and wheat will be attempted.

We observed no significant change in the percentage of wheat plants showing loose smut symptoms caused by *U. nuda* f. sp. *tritici*, although the infection level fluctuated over the years, from 5.2% at the start, increasing to 9.0% after 10 years, and declining to 4.7% after 30 years (Table 2). Previous studies have shown both increased and decreased percentage of smutted plants grown from seeds in long-term storage. A Canadian study showed an increased proportion of smutted plants (from 56 to 91%) over 20 years of wheat seed storage at −15 °C and low relative humidity [35]. A previous study in barley reported 73% smutted heads after 7 years of storage at 0 °C [36]. On the other hand, the percentage of barley plants infected with *U. nuda* in the field declined from 14% initially to only 3.2% at the end of an 11-year period when the seed was stored at room temperature [37].

We detected low infection frequencies of *A. radicina* (causing black root rot and damping-off) and *A. dauci* (causing leaf blight and damping-off) in carrot at the start of the experiment, and despite some variation during the years no trends in the infection levels were found (Table 2). Likewise, no decline was observed in the infection percentage of *A. dauci* (from 22% to 21%) and only a slight reduction in *A. radicina* (from 37% to 28%) after carrot seeds were stored for 14 years at −20 °C in the United Kingdom [23].

Despite low initial infection levels, *B. allii* (causing neck rot in onion) survived after 30 years of storage. An increase in the infection level was observed after 10 to 20 years of storage, though, a slight reduction was observed at the two last testing events (Table 2). One reason for the increased infection percentage might have been the difficulty in accurately identifying *Botrytis* spp. Confusing *B. allii* with the very common *B. cinerea*, and possibly *B. squamosa*, which can also occur in onion seed [38], might have led to an overestimation of the *B. allii* infection percentage. A study from the United Kingdom found internal seed infections of *B. allii* to persist for 3.5 years in onion seeds (20% infection) stored at 10 °C and 50% relative humidity [39].

The infection frequency of LMV remained stable from 1.8% at the start of the experiment to 2.0% after 30 years. We did not find any literature on the survival of this virus; however, other embryo-borne viruses are known to survive for several years, as described by Neergaard [6].

### 3.4. Survival of S. sclerotiorum Sclerotia

The viability of *S. sclerotiorum* sclerotia, confirmed as mycelial growth on agar plates, varied considerable over the years, from 8 (40%) to 20 (100%) of the 20 sclerotia plated at each time point. Some sclerotia were totally or partly damaged by saprophytes which might be an indicator of death and degradation of the sclerotia. Nevertheless, we conclude that dry sclerotia can survive for at least 30 years at below-zero temperatures. This is not surprising because sclerotia are surviving structures and they are known to survive for several years, both through contamination of seed lots and in the soil [40].

## 4. Conclusions

So far, all seed-borne pathogens included in the 100-year seed storage experiment have survived, and only a few of them have shown a reduction in the infection percentages during the first 30 years. Our study is limited to only one seed lot of each pathogen/host species combination, each representing an example of longevity of seed-borne pathogens. Although all seed samples were stored at the same conditions (3–5% moisture content in the seeds, –3.5 °C), the survival of the pathogens is influenced by several other factors such as host genotype, location of inoculum in the seeds, and type of surviving structures, as mentioned previously. These factors were not known for the included material. Nevertheless, we believe our study adds new and interesting information on the survival of pathogens during seed storage. We showed that crops commonly grown in Nordic countries can host seed-borne pathogens for a long time when dry seeds are stored at low or below-zero temperatures. The longevity of seed-borne pathogens during such conditions emphasizes the importance of maintaining high phytosanitary standards in seed gene banks and implementing routines that avoid the use of infected seeds and spread of diseases.

## Figures and Tables

**Table 1:** Host crop species, cultivars (and country of origin), pathogen species studied, and the analysis methods used in the 100-year seed storage experiment.

Host Crop Species, Cultivar (Country of Origin)	Pathogen Species ^1^	Analysis Methods ^3,4^
Wheat 1 (*Triticum aestivum*) ‘Runar’ (Norway)	*Septoria nodorum* *Fusarium* spp.	FBM [11,12,13]
Wheat 2 (*Triticum aestivum*) ‘Line 79 CBW A 72′ (Canada)	*Ustilago nuda* f.sp. *tritici*	SM
Barley (*Hordeum vulgare*) ‘Bamse’ (Norway)	*Drechslera* spp. *Fusarium* spp.	FBM [11,12]
Meadow fescue (*Festuca pratensis*) ‘Salten’ (Norway)	*Drechslera dictyoides*	FBM [11,12]
Timothy (*Phleum pratense*) ‘Forus’ (Norway)	*Drechslera phlei*	FBM [11,12]
Lettuce (*Lactuca sativa*) ‘Attractie’ (The Netherlands)	Lettuce mosaic virus (LMV)	SM [14] + ELISA [15]
Onion (*Allium cepa*) ‘Laskala’ (Norway)	*Botrytis allii* *Fusarium* spp.	FBM [11,12]
Carrot (*Daucus carota*) ‘Forto Nantes’ (the Netherlands)	*Alternaria radicina* *Alternaria dauci*	FBM [11,12,16,17]
Beet (*Beta vulgaris*) ‘Hilma’ (United Kingdom)	*Phoma betae*	WA [18]
Cabbage (*Brassica oleracea* ssp. *capitata* f. *alba*) ‘Trønder Lunde’ (Norway)	*Alternaria brassicicola*	FBM [11,12,19]
(Norway)	*Sclerotinia sclerotiorum* ^2^	PDA

^1^ Although the taxonomic nomenclature has been updated with new names for some of the pathogens, we have maintained the scientific names which were given in the instructions with the samples in the storage boxes. ^2^ 1 sample of *Sclerotinia sclerotiorum* sclerotia, collected from a Norwegian cabbage (*Brassica oleracea*) seed lot and conserved separately in the ampoules without any seed material, was included. ^3^ FBM = Freezing blotter method, SM = Symptom method (‘growing-on’ test in greenhouse), ELISA = Enzyme linked immunosorbent assay, WA = Water agar, PDA = Potato dextrose agar. ^4^ Numbers in brackets are references.

**Table 2:** Percentages of infected seeds and simple linear regression analyses of pathogen infection during the first 30 years of the 100-year storage experiment with 10 seed lots stored at −3.5 °C (permafrost) in an abandoned coalmine in Svalbard, Norway.

Crop Species	Pathogen	Storage Years											Regression Equation	R^2^	*p*-Value ^1^
		0	2.5	5	7.5	10	12.5	15	17.5	20	25	30			
Wheat 1	*Septoria nodorum*	15	16	13	4	11	9	5	3	13	7	1	y = 13.7 − 0.4x	45.8	0.02
	*Fusarium* spp.	5	10	12	1	6	9	8	2	4	6	7	y = 7.3 − 0.1x	4.0	0.56
Wheat 2	*Ustilago nuda* f.sp. *tritici*	5.2	5.7	7.7	7.2	9.0	7.7	8.4	7.0	5.2	4.9	4.7	y = 7.3 − 0.0x	11.8	0.30
Barley	*Drechslera* spp.	38	36	64	45	50	41	36	50	54	30	47	y = 45.5 − 0.1x	0.4	0.86
	*Fusarium* spp.	8	13	8	2	22	8	7	5	10	18	8	y = 9.1 + 0.1x	1.0	0.77
Meadow fescue	*Drechslera dictyoides*	63	62	61	47	51	57	47	52	47	37	34	y = 62.9 − 0.9x	81.1	0.00
Timothy	*Drechslera phlei*	70	77	69	71	76	73	66	65	65	63	65	y = 73.9 − 0.4x	51.8	0.01
Lettuce	Lettuce mosaic virus (LMV)	1.8	3.8	4.0	3.0	2.4	2.9	2.2	2.4	1.9	1.7	2.0	y = 3.2 − 0.0x	33.2	0.06
Onion	*Botrytis allii*	4	4	3	5	10	13	21	7	10	2	1	y = 7.5 − 0.0x	0.1	0.95
	*Fusarium* spp.	1	1	1	1	1	4	1	0	1	1	4	y = 0.8 + 0.0x	13.1	0.27
Carrot	*Alternaria radicina*	4	2	12	8	2	8	5	5	12	4	6	y = 5.7 + 0.0x	0.9	0.78
	*Alternaria dauci*	6	9	9	12	10	12	12	15	12	6	9	y = 9.7 + 0.0x	1.7	0.70
Beet	*Phoma betae*	43	36	45	36	38	49	41	53	55	44	56	y = 38.3 + 0.5	45.0	0.02
Cabbage	*Alternaria brassicicola*	90	88	92	98	95	98	97	99	93	82	84	y = 95.1 − 0.2x	11.4	0.31

^1^ *p*-value < 0.05 indicates a significant trend during the years (tested by a simple regression model).

## Data Availability

Not applicable.
